# Origins of 1/*f *noise in nanostructure inclusion polymorphous silicon films

**DOI:** 10.1186/1556-276X-6-281

**Published:** 2011-04-04

**Authors:** Shibin Li, Yadong Jiang, Zhiming Wu, Jiang Wu, Zhihua Ying, Zhiming Wang, Wei Li, Gregory Salamo

**Affiliations:** 1 State Key Laboratory of Electronic Thin Films and Integrated Devices, School of Optoelectronic Information, University of Electronic Science and Technology of China (UESTC), Chengdu 610054, China; 2 Arkansas Institute for Nanoscale Materials Science and Engineering, University of Arkansas, Fayetteville, AR 72701, USA; 3 Department of Electronics and Information, Hang Zhou Dianzi University, Hangzhou, 310018, China

## Abstract

In this article, we report that the origins of 1/*f *noise in *pm*-Si:H film resistors are inhomogeneity and defective structure. The results obtained are consistent with Hooge's formula, where the noise parameter, *α*_H_, is independent of doping ratio. The 1/*f *noise power spectral density and noise parameter *α*_H _are proportional to the squared value of temperature coefficient of resistance (TCR). The resistivity and TCR of *pm*-Si:H film resistor were obtained through linear current-voltage measurement. The 1/*f *noise, measured by a custom-built noise spectroscopy system, shows that the power spectral density is a function of both doping ratio and temperature.

## Introduction

Nanostructure semiconductor has been the focus of intense interest in recent years due to their extensive device application [1-6]. It is well known that hydrogenated polymorphous silicon is a nanostructure inclusion material [7-9]. Hydrogenated silicon films commonly exhibit high noise at low frequency (*f*). This noise has a spectral power density of the type *S*(*f*) ∝ 1/*f*^*a*^, where *a *is known as "1/*f *noise." However, lower noise materials are important for high-performance semiconductor devices. 1/*f *noise of amorphous and polycrystalline silicon has captured the attention of researchers in the field of electronics and physics for several decades [10]. Polymorphous silicon film is generally prepared by operating a strong hydrogen-diluted silane plasma source at high pressure and power density [11]. Many efforts have been made concerning the growth process, microstructure, transport, and optoelectronic properties of *pm*-Si:H films [12]. The results indicate that *pm*-Si:H films show higher transport properties than *a*-Si:H, a highly desirable trait for the production of devices, such as solar cells and thin film transistors. To date, *pm*-Si:H investigations have focused on certain applications, but there is no study devoted to the 1/*f *noise of such materials except those by our group which have reported the dependence of 1/*f *noise on the change of material structure of silicon films [13-15]. In this article, we focus on the study of the origins of 1/*f *noise in *pm*-Si:H and investigate the influence of boron doping ratio on 1/*f *noise in *pm*-Si:H films.

## Experimental

**Figure 1:**
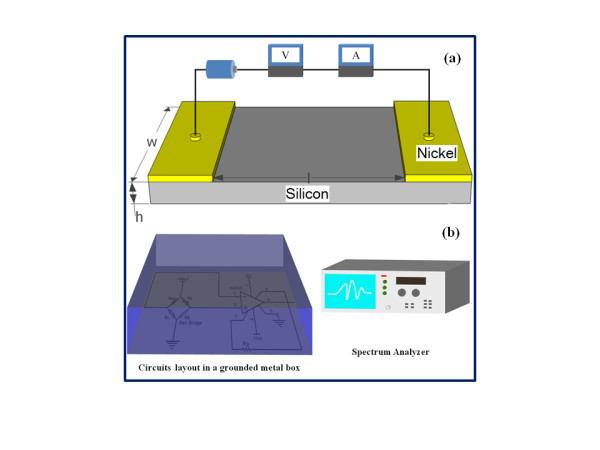
**Schematic of measurement system**. **(a) **Schematic of coplanar electrode configuration for thin *pm*-Si:H film resistance measurement; **(b) **schematic diagram of low-frequency noise measurement system.

The *pm*-Si:H films were obtained by using RF PECVD [11]. As shown in Figure 1a, Coplanar nickel electrodes (about 50 nm) were evaporated onto the *pm*-Si:H films and lifted off to make linear *I*-*V *contact. In Figure 1b, in order to reduce external noise disturbance, the measuring circuit was placed in a metal box. The noise and electrical measurements were performed at various temperatures using an ESL-02KA thermostat. Hall measurements were performed using a BioRad HL5560 Hall system coupled with helium cryostat. The structure of *pm*-Si:H films was characterized using a SE850 spectroscopic ellipsometer with Bruggeman effective medium model.

## Results and discussions

**Figure 2:**
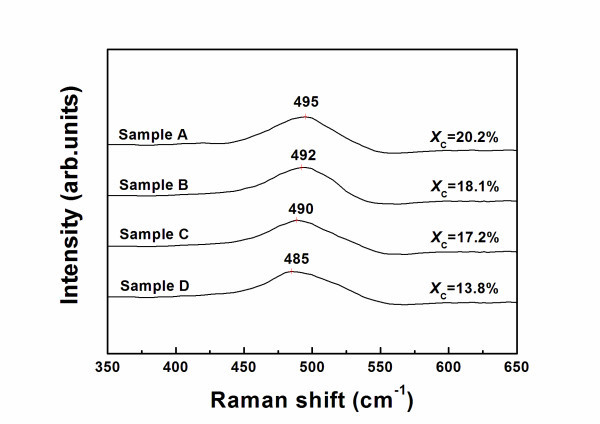
**Raman spectroscopy of polymorphous silicon samples**. Raman spectroscopy for *pm*-Si:H samples (A, B, C, D), the crystal volume fractions *X*_C _(%) obtained by Raman is consistent with the results from SE measurements.

The results in Table 1 show that *pm*-Si:H films deposited at higher doping ratio were characterized by high hydrogen content and crystalline fraction, and negligible void fraction. As shown in Figure 2, because of its nanocrystalline nature, the crystalline Raman peak of *pm*-Si:H exhibits a frequency downshift and peak broadening caused by a phonon confinement effect. A peak (*I*_n_) is observed between 480 cm^-1 ^(*I*_a_: amorphous silicon) and 520 cm^-1 ^(*I*_c_: microcrystalline silicon). The crystalline volume fraction *X*_C _of these films has been calculated from the relation *X*_C _= (*I*_n _+ *I*_c_)/(*I*_a _+ *I*_n _+ *I*_c_) [13]. In this study, the results have proven that the crystalline volume fractions (*X*_C_) measured by SE and Raman spectroscopy are highly consistent.

**Table 1:** Structure and electrical properties for different doping ratios in *pm*-Si:H film

Sample	***R***_**v**_	*h *(nm)	***R***_**0 **_**(MΩ)**	***N***_**C**_	*β *(%)	***X***_**C **_**(%)**	*H *(%)	Void (%)
A	10^-2^	326	2.42	4.24 × 10^14^	2.78	20	18	0
B	10^-3^	335	4.53	3.54 × 10^13^	2.93	18	16	0.5
C	10^-4^	341	6.76	4.23 × 10^12^	3.28	17	15	0.8
D	10^-5^	352	8.21	3.85 × 10^11^	3.45	14	13	1

*R*_v_, gas doping ratio; *h*, film thickness (nm); *N*_C_, total number of carriers; TCR value-*β *(%), TCR = 1/R*(*dR*/*dT*)*100; resistivity at temperature of 300 K-*R*_0 _(MΩ); *X*_C_, crystal volume fraction (%); H, hydrogen content (%); void, void volume fraction (%)

**Figure 3:**
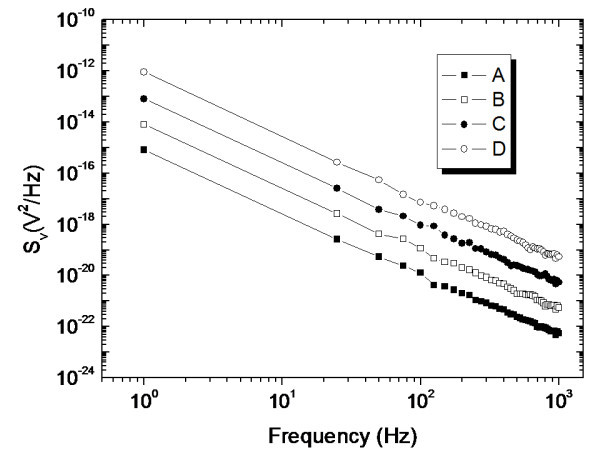
**Log-log plot of power spectra density for various doping ratios in *pm*-films at 50 mV bias**.

Figure 3 shows a logarithmic plot of power spectral density, which is averaged over 30 measurements, versus frequency for different doping ratios in *pm*-Si:H films at 300 K. The decrease of noise is inversely proportional to frequency. Moreover, the 1/*f *noise decreased with the increment of boron doping ratio in *pm*-Si:H samples. Conventionally, the results of 1/*f *noise measurements are discussed using Equation 1 originally introduced by Hooge [16]:(1)

where *S*_v _is the noise power density at voltage *V*, *α*_H _is the noise parameter, *f *is frequency, and *N*_C _is the total number of charge carriers in a certain volume involved in noise generation. The total number of charge carriers, determined by Hall measurement, in conjunction with the dimension of the *pm*-Si:H film resistor, determines the noise parameter *α*_H _as a function of frequency. Our experimental results also demonstrate the 1/*f *noise power scales with the square of bias voltage, which is in agreement with the results of Fine et al. [17].

**Figure 4:**
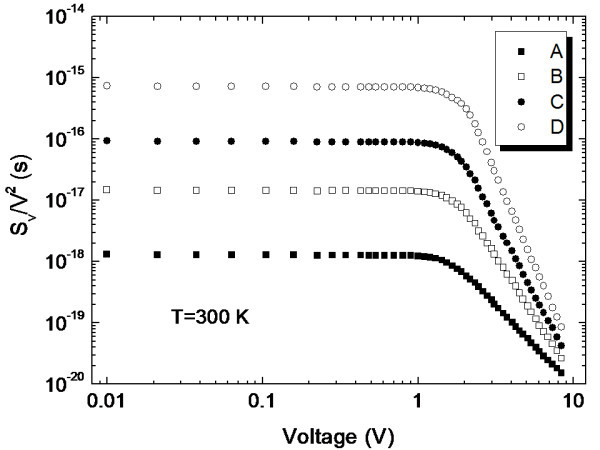
**Relative noise power at 100 Hz vs voltage**. Relative noise power demonstrates the dependence of 1/*f *noise in silicon film on structure variation.

Figure 4 shows the relative voltage noise power *S*_v_/*V*^2 ^at 100 Hz. We obtained that *S*_v_/*V*^2 ^is constant at voltage less than 1 V, which indicates that 1/*f *noise in *pm*-Si:H film resistor does not originate from the resistance fluctuations at 100 Hz under our experimental conditions. *Pm*-Si:H film is generally accepted as inclusion material in nanocrystalline and nanosized clusters [18]. The above results indicate that *pm*-Si:H films are far from being homogeneous, and thus, one could predict that their electronic properties are affected by heterogeneity. For the clarification of our results, the structure and 1/*f *noise variations in amorphous, microcrystalline, and *pm*-Si:H films were compared [13]. The results demonstrate the dependence of 1/*f *noise in silicon film on the structure variation. Paul and Dijkhuis [19] proved the influence of metastable defect creation on the noise intensity in hydrogenated amorphous silicon. Hence, we also believe that the defects and heterogeneity cause 1/*f *noise in *pm*-Si:H.

**Figure 5:**
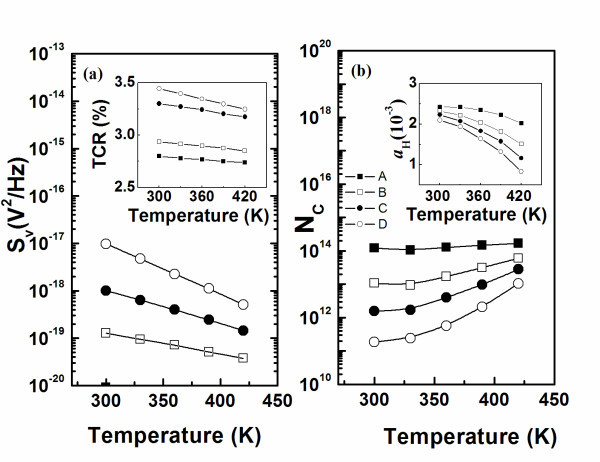
**Temperature dependence of 1/*f *noise in *pm*-Si:H film**. **(a) **Temperature dependence of 1/*f *noise in *pm*-Si:H film. Inset: temperature dependence of TCR value for samples with various doping ratios; **(b) **temperature dependence of total carriers number (*N*_C_) on various doping ratios in *pm*-Si:H films. Inset: temperature dependence of noise parameter in Hooge's formula.

The temperature dependence of 1/*f *noise in *pm*-Si:H film resistor was also measured at 100 Hz for the various boron doping *pm*-Si:H film resistors at temperatures ranging from 300 to 420 K. In Figure 5a, the 1/*f *noise in *pm*-Si:H film resistor decreases with the increasing temperature. From the theoretical model proposed by Richard, there is a correlation between *S*_v _and the temperature coefficient of resistance (TCR) given by Equation 2 [20]:(2)

where  is the average voltage biased on the sample, 〈(Δ*T*)^2^〉 is mean-square temperature fluctuation, and *β *is the value of TCR [13]. In the case of our measurement condition, the value of  and 〈(Δ*T*)^2^〉 is the same for each film resistor. Therefore, the power spectral density of 1/*f *noise in *pm*-Si:H film resistors is proportional to squared *β *(*S*_v_(*f*) ∝ β^2^). The TCR is a function of resistivity in *pm*-Si:H film resistors, which means that resistance fluctuation is another origin of 1/*f *noise in the *pm*-Si:H resistors when the measurement temperature changed significantly. Figure 5b shows that the temperature dependence of the total charge carrier number in the measured volume also decreases with increasing boron doping ratio. The more highly doped the sample (such as sample A) the fewer the dangling bonds and defects. Therefore, the variation in the total charge carrier number for the higher-doped *pm*-Si:H sample is lower. From Equation 1, we obtain(3)

For each measured sample here, the values of *N*_C_, *f*, and *V*^2 ^are constant. The value of noise parameter *α*_H _at 100 Hz is plotted against temperature for different doping ratios as shown in the inset of Figure 5b. The noise parameter *α*_H _for the *pm*-Si:H film resistors in this study is also a function of the squared TCR (α_H _∝ β^2^). It demonstrated that the resistance fluctuation of the film samples also resulted in the variation of noise parameter when the measurement temperature changed dramatically.

## Conclusions

The results of this study demonstrated that the origins of 1/*f *noise in nanostructure inclusion *pm*-Si:H are the inhomogeneity and the defective structure in the films. The power spectral density of 1/*f *noise is inversely proportional to boron doping ratio, which is consistent with Hooge's formula. The value of *S*_v_/*V*^2 ^is constant when the voltage is less than 1 V, demonstrating that resistance fluctuation is not the origin of 1/*f *noise in *pm*-Si film resistors in the case of constant temperature. At 100 Hz, the temperature dependence of 1/*f *noise indicates that the power spectral density and the noise parameter *α*_H _are proportional to the squared TCR. It has also been proven that the resistance fluctuation of the film samples also results in the variation of noise parameter when the measurement temperature changed dramatically.

## Abbreviations

TCR: temperature coefficient of resistance.

## Competing interests

The authors declare that they have no competing interests.

## Authors' contributions

SL designed the experiments, carried out the sample preparation and 1/*f *noise measurement. JW and ZY worked on organize data. All authors participated in discussion on writing. All authors read and approved the final manuscript.
